# Colorectal Cancer: How Familiar Are Our Future Doctors with the Cancer of Tomorrow?

**DOI:** 10.1155/2018/7462101

**Published:** 2018-06-05

**Authors:** Vaman Kulkarni, B. B. Darshan, Bhaskaran Unnikrishnan, Kho Chun Cheng, Goh Cia Hui, Ang Yee Theng, Kong Sik Yuien, Rekha Thapar, Prasanna Mithra, Nithin Kumar, Ramesh Holla, Avinash Kumar

**Affiliations:** Department of Community Medicine, Kasturba Medical College (Affiliated to Manipal Academy of Higher Education), Mangalore 575001, India

## Abstract

**Background:**

Colorectal cancer (CRC) is one of the common cancers affecting both genders. Although the incidence of CRC is low in India there has been an increase in the past few decades.

**Objective:**

To assess the awareness regarding colorectal cancer and its screening among medical students and interns.

**Methods:**

This cross-sectional study was conducted among 290 participants (final year medical students and interns) from Kasturba Medical College, Mangalore. A pretested semistructured questionnaire was used to collect information. Data was analyzed using SPSS 17.0.

**Results:**

Majority of participants had satisfactory knowledge regarding CRC. 38% of them scored excellently, 64.8% had good knowledge, and 5.2% scored poorly. Knowledge regarding CRC symptoms was good (95%). 92% of the participants were aware of risk factors of CRC. Only 49% of the participants identified FOBT as a screening tool and 30.7% participants knew that 50 years is the recommended age to begin CRC screening. Interns and international students had better knowledge than final year medical students and Indian students and this was found to be statistically significant.

**Conclusion:**

There is a need to improve participant's knowledge regarding CRC screening although majority of them are aware of CRC symptoms and risk factors.

## 1. Introduction

Globally, colorectal cancer (CRC) is the third most common cancer in men (10.0% of the total cancers) and the second in women (9.4% of the total cases) [1]. It is estimated that CRC causes 26,270 male and 24,040 female deaths worldwide [2]. The highest rates are estimated to be in New Zealand/Australia and western part of Europe and the lowest in Africa (excluding Southern Africa) and South-Central Asia [1].

Incidence rates differ widely within Asian countries and the number of CRC cases has increased drastically in certain economically mature parts of Asia. There is an increase of 2-4 times in the incidence of colorectal cancer in the past few decades [1, 3, 4]. The rising pattern in incidence and mortality from colorectal cancer is more in the affluent as compared to the poorer communities [5].

Compared to the western world, the age-adjusted incidence rates of colorectal cancer are low in India as recorded by all cancer registries in India [3]. The incidence of colon cancer is found to be 0.7 to 3.7/100,000 among men and 0.4 to 3/100,000 among women whereas rectal cancer incidence is 1.6 to 5.5/100,000 among men and 0 to 2.8/100,000 among women [4]. Despite this, an increase in CRC incidence rate is foreseeable in India due to increase in urbanization and rapid changes in lifestyle among the population of the country [1].

Colorectal cancer symptoms often do not present themselves until the disease has progressed to the advanced stages, leading to the cancer being diagnosed after the development of symptoms [6].

The common symptoms of CRC are altered bowel habits, rectal bleeding, constipation, diarrhoea, unexplained weight loss, etc. with rectal bleeding being the most important symptom [7].

There is compelling evidence to support screening moderate-risk individuals above the age 50 years to detect and prevent CRC [8]. Screening can lower CRC mortality by identifying cancer at an early, curable stage and by detecting and removing clinically significant adenomas [9]. CRC also has a long preclinical phase and the survival of patients whose disease is detected at an early stage is favorable [10]. Screening tests for CRC broadly fall into two categories. Category 1 consists of the faecal tests, which includes Faecal Occult Blood Testing (FOBT), Faecal Immunohistochemical Testing (FIT), and sDNA. Flexible sigmoidoscopy, colonoscopy, double-contrast barium enema (DCBE), and computed tomography colonography (CTC) belong in Category 2 [11]. Among these, FOBT remains a valuable screening tool [12]. Although CTC is widely accepted in western population, it is rarely used in India due to technical limitations [13].

A scant amount of research has been done to assess colorectal cancer awareness levels within the Indian population. CRC, although now a minor disease in India, will continue to prevail and incidence rates will increase in conjunction with the country's development. It is thus vital that a practicing physician possesses the required and expected knowledge to counsel patients regarding CRC and its screening. Therefore through our study, we intend to assess the awareness and knowledge regarding colorectal cancer and its screening among students and interns of a medical college in Mangalore.

## 2. Materials and Methods

The present cross-sectional study was conducted among medical students and interns of Kasturba Medical College, Mangalore, using convenience sampling technique. For the purpose of the study, a medical student is defined as the one who is studying in one of the nine semesters of Bachelor of Medicine and Bachelor of Surgery (MBBS) course. We included students from final year considering the fact that the surgical theory and clinical exposure is more extensive during this phase compared to earlier phases of MBBS. An intern is defined as the trainee who has finished MBBS and is now working in the hospitals attached to KMC, Mangalore, under the supervision of senior faculty members and postgraduates. Considering the knowledge regarding CRC among medical students at 69% [8], with a power of 80%, absolute precision of 6%, and confidence level of 95% and a nonresponse rate of 10%, a sample size of 290 was calculated. Approval was obtained from Institutional Ethics Committee (IEC) of Kasturba Medical College, Mangalore. The study was conducted after obtaining the informed consent from participants which included equal number of final year medical students and interns. The study was conducted over a period of two months (March-April 2015). The pretested semistructured questionnaire consisted of four parts. The first part consisted of sociodemographic factors. The second part contained questions regarding awareness of CRC and its clinical features (clinical symptoms and risk factors of CRC). In the third part, questions were asked regarding screening of CRC and the fourth part about need for further knowledge regarding CRC. A scoring system was designed to grade their knowledge regarding CRC and its screening. Twenty-three questions were selected for scoring. Each question carried an equal weightage of 1 mark. One mark was given to each correct answer and no marks were given to wrong or unsure answers. Scores 0-10 were graded as poor; scores 11-17 were graded as good; and scores 18-23 were graded as excellent. As a part of the study, the participants were given pamphlets after completing the questionnaire. The pamphlets provided some information regarding CRC and its screening** (Annexure 1)**. Data was entered and analyzed using SPSS version 17. The descriptive statistics were done in terms of percentages, means, medians, etc. Univariate analysis was done using Chi-square test.

## 3. Results

A total of 290 participants were included in our study. Among them, 125 participants (43.1%) were males and 165 (56.9%) were females. There was an equal distribution of final year medical students and interns of 145 (50%), respectively. Majority of participants were Indians (n = 258, 89%) while the rest were international students (n = 32, 11.0%). The mean age of the participants was 22.35 years, with a range of 20-26 years.

The awareness of CRC incidence rates in India among participants was unsatisfactory. Half of the participants felt that the CRC incidence rate in India is on a moderate scale. Only, n = 52, 18.0%, correctly responded that the CRC incidence rate is low. Additionally, n = 70, 24% participants assumed that CRC incidence rate is high in India while n = 23, 8%, of them were unsure.

Table 1 depicts students' response regarding symptoms and risk factors regarding CRC. Majority of the students responded that rectal bleeding (n = 278, 95.9%) and altered bowel habits are the most common symptoms. Also family history of CRC (n = 268, 92.4%) and inflammatory bowel disease (n = 260, 89.7%) were given as answers by them for the major risk factors of CRC.

Participants were found to be less informed about the recommended age to begin CRC screening. Only, n = 89, 30.7% participants answered that 50 years was the recommended age for screening.

When the participants were asked regarding methods for screening of CRC, more than two-third of them (n = 195, 67.2%) responded that flexible sigmoidoscopy is the major screening test followed by colonoscopy (n = 192, 66.2%) as noted in Table 2. However very few (n = 87, 30%) participants received any information regarding CRC. Nonetheless majority of them were willing to receive any further information provided to them regarding CRC (n = 251, 86.6%) (Table 2).

Table 3 observes that the major source of knowledge regarding CRC was textbooks (n = 269, 92.8%) followed by classroom teaching (n = 220, 75.9%). Only 22.4% (n = 65) participants received information from friends or family.

The participant's knowledge levels regarding CRC was further categorized into excellent, good, and poor based on the criteria mentioned in the methods section. Majority of the participants had satisfactory knowledge regarding CRC and its screening. Thirty percent (n = 87) of participants scored excellently, whereas 64.8% (n = 188) had good knowledge. Only 5.2% scored poorly (n = 15).

We grouped the study participants into the following categories: interns and final year students, Indian and international students, and males and females. Univariate analysis to assess the difference in knowledge levels among these groups revealed that interns had higher knowledge compared to final year students (*p* = 0.01) and international students had better knowledge compared to Indian students (*p* = 0.021). Male participants had better knowledge than females; however the difference was not statistically significant (*p* = 0.937) (Table 4).

## 4. Discussion

This study was conducted with an objective to assess the knowledge level of final year medical students and interns of a medical college in Mangalore, India, regarding CRC and its screening.

Although CRC incidence rates are low in India [3], half of the participants chose medium as their response. Even though the incidence of CRC is low in India, it is important for the future doctors to be aware of the epidemiology of CRC as there is a possibility of increase in its burden in near future.

Majority of the participants had satisfactory knowledge regarding CRC and its screening. Thirty percent of participants scored excellently whereas 64.8% had good knowledge. Only 5.2% scored poorly. This finding is on par with the results obtained from a study done in Greece and Malaysia [14, 15]. This finding is of importance since higher knowledge levels regarding CRC are essential in its early detection and treatment.

More interns had scored excellently (37.2%) compared to final year medical students (22.8%). Overall, interns performed better than final year students. Similar phenomenon was observed in a study in Mexico, where internal medicine residents possessed better knowledge regarding cancer screening compared to medical students [16]. This finding is probably due to greater exposure to various clinical cases among interns who have already started working in the hospitals. Besides that, international students displayed better knowledge and awareness (50%) compared to Indian students (27.5%). Higher CRC incidence rates in their countries of origin may be a contributing factor to this.

Overall, the participants were knowledgeable on the symptoms and risk factors of CRC. More than 90% of the participants recognized rectal bleeding, altered bowel habits, and unexplained weight loss as the symptoms of CRC. This result is consistent with that of a similar study carried out among Greek medical students, in which 85-99% of the medical students had successfully identified the symptoms of CRC [14]. On the contrary, studies conducted elsewhere reported a low knowledge regarding CRC symptoms [17–20]. However, a comparatively few number of participants of this study (54.1%) identified diarrhoea as a symptom while only 27.6% have included diabetes as a risk factor despite diabetes being significantly associated with fatal colon cancer in a study done on US adults [21] and diarrhoea being established as a clinical feature of CRC before diagnosis [6]. It is important for the doctors of tomorrow to be aware of all the clinical features and risk factors of CRC in absence of which the prevention and control of its morbidity and mortality will be severely affected.

It is encouraging to note that most of the participants (88.6%) responded that CRC screening plays an important role in the prevention of death and disabilities. This can be attributed to the fact that the participants as medical professionals realized the importance of disease screening as a preventive measure. In contrast, studies conducted in Singapore and Hong Kong showed that only a minority of the respondents were aware of screening as an essential means against developing CRC [17, 22]. In terms of recommended age for the initiation of CRC screening, only 30.7% of the participants had given the correct answer. In comparison, Greek medical students performed better (83%) regarding the age of initiation of CRC screening [14]. The knowledge regarding proper age of initiation of screening for CRC is of paramount importance because delay in detection may lead to increased burden of advanced CRC cases in the community.

Majority of the participants (66%) were aware that flexible sigmoidoscopy and colonoscopy are a part of the screening tests while only 49% of them responded FOBT as a screening tool. In contrast, the knowledge levels were lower among studies conducted elsewhere [15, 22, 23]. This finding is of importance as the awareness regarding tools for screening CRC is essential in early detection of CRC and thereby it will help in reducing the morbidity.

A large number of participants (66.6%) had not received any information regarding CRC screening prior to this study. About 80% of the participants were inexperienced in advising and encouraging others to undergo colonoscopy. An encouraging number of participants (86.6%) had expressed their willingness to receive further information and this percentage is relatively high compared to the reported 68% among the Greek medical students [14]. Majority of the participants (80.7%) are keen on informing family and friends about screening benefits, perhaps foreseeing an increase of CRC cases in the future. This is very important, as a study has shown that social influence plays a major role in affecting screening behavior of Singaporean-Chinese, in which 67.5% agreed to go for CRC screening if their family wanted them to [24].

The limitations of the present study include the following: since the present study was conducted in a single setup, the findings cannot be extrapolated to other settings. A multicentric study could have provided a better evidence. Nonetheless, our study was conducted using adequate sample size and had better response rates and also the research was followed up with provision of information regarding CRC in the form of educative pamphlets.

## 5. Conclusion

In conclusion, the current study demonstrated an overall higher knowledge regarding CRC among the young future doctors from India. However, crucial aspects of CRC prevention and control, such as age at screening and modalities of screening, were known to few of the participants. In addition, the participants did not receive any additional information regarding CRC other than their academic exposure and were in favor of additional information in case it is provided. Hence, further awareness generation through activities like seminars, workshops, etc. would go a long way in addressing this cancer of future in India.

## Figures and Tables

**Table 1:** Knowledge regarding CRC symptoms and risk factors (n = 290).

**Component**	**Response**		
	**Yes**	**No**	**Unsure**
	N (%)	N (%)	N (%)
**Which among the following are symptoms of CRC**			
Rectal Bleeding	278 (95.9)	004 (01.4)	008 (02.7)
Altered Bowel Habits	275 (94.8)	005 (01.7)	010 (03.5)
Constipation	208 (71.7)	023 (07.9)	059 (20.4)
Diarrhea	157 (54.1)	050 (17.2)	083 (28.7)
Unexplained weight Loss	272 (93.8)	007 (02.4)	011 (03.8)

**Which among the following are risk Factor of CRC**			
Old Age	211 (72.8)	019 (06.6)	060 (20.6)
Inflammatory Bowel Disease (Ulcerative Colitis)	260 (89.7)	013 (04.5)	017 (05.9)
Family History of CRC	268 (92.4)	005 (01.7)	017 (05.9)
Family History of Colon Polyps	260 (89.7)	011 (03.8)	019 (06.5)
Low Fiber diet	257 (88.6)	010 (03.4)	023 (07.9)
High Fat Diet	184 (63.4)	052 (17.9)	054 (18.6)
Sedentary Life style	157 (54.1)	049 (16.9)	084 (29.0)
Diabetes	080 (27.6)	078 (26.9)	132 (45.5)
Obesity	174 (60.0)	035 (12.1)	081 (27.9)
Smoking	217 (74.8)	031 (10.7)	042 (14.5)
Heavy alcohol consumption	176 (60.7)	030 (10.3)	084 (29.0)

**Table 2:** Knowledge regarding CRC screening (N = 290).

**Comment **	**Response**		
	**Yes**	**No**	**Unsure**
	**N (%)**	**N (%)**	**N (%)**
**Knowledge regarding CRC screening**			
Screening prevents deaths and disabilities	257 (88.6)	009 (3.1)	024 (08.3)
Possibility of removal of polyps with colonoscopy	214 (73.0)	037 (12.8)	003 (13.4)

**Screening tool**			
Faecal Occult Blood Testing (FOBT)	142 (49.0)	033 (11.4)	115 (39.7)
Flexible sigmoidoscopy	195 (67.2)	010 (03.4)	085 (29.3)
Colonoscopy	192 (66.2)	013 (04.5)	085 (29.3)
Have you received information	087 (30.0)	193 (66.6)	010 (03.4)
Willingness to receive information	251 (86.6)	026 (09.0)	013 (04.5)

**Table 3:** Chief sources of knowledge regarding CRC (N = 290).

**Source **	**Response**		
	**Yes**	**No**	**Unsure**
	**N (%)**	**N (%)**	**N (%)**
Textbooks	269 (92.8)	021 (07.2)	00
Classroom teaching	220 (75.9)	070 (24.1)	00
Internet	154 (53.1)	136 (46.9)	00
Friends/Family	065 (22.4)	225 (77.6)	00
Colonoscopy Advice & Encouragement	059 (20.3)	230 (79.3)	001 (00.3)
Colonoscopy among family members	060 (20.7)	227 (78.3)	003 (01.0)
Informing family & friends regarding screening benefits	234 (80.7)	034 (11.7)	022 (07.6)

**Table 4:** Association between level of study, nationality, gender, and knowledge regarding CRC (N = 290).

	**Excellent**	**Good**	**Poor**	***P* value**
	**N (%)**	**N (%)**	**N (%)**	
**Level of study**				
Medical students	33 (22.8)	099 (68.3)	013 (09.0)	**0.01**
Interns	54 (37.2)	089 (61.4)	002 (01.4)	

**Nationality**				
Indian	71 (27.5)	174 (67.4)	013 (05.0)	**0.021**
Others	16 (50.0)	014 (43.8)	002 (06.2)	

**Gender**				
Male	38 (30.4)	080 (64.0)	007 (05.6)	**0.937**
Female	49 (29.7)	108 (65.5)	008 (04.8)	
